# Color Variation and Secondary Metabolites’ Footprint in a Taxonomic Complex of *Phyteuma* sp. (Campanulaceae)

**DOI:** 10.3390/plants11212894

**Published:** 2022-10-28

**Authors:** Mariana Cecilia Grohar, Aljaz Medic, Tea Ivancic, Robert Veberic, Jernej Jogan

**Affiliations:** 1 Department of Agronomy, Biotechnical Faculty, University of Ljubljana, Jamnikarjeva ulica 101, 1000 Ljubljana, Slovenia; 2 Department of Biology, Biotechnical Faculty, University of Ljubljana, Vecna pot 111, 1000 Ljubljana, Slovenia

**Keywords:** phenolic compounds, anthocyanins, phenolic acids, flavonols, HPLC-MS, identification, Slovenia

## Abstract

In the genus *Phyteuma*, the taxonomic delimitation of some species is difficult since a high variability of morphological traits, such as flower color, is present, probably due to high levels of hybridization. Historic descriptions and the morphological traits used in the taxonomic keys are sometimes unclear and lead to misinterpretations. Here, a detailed analysis of flower color variability in different populations of sympatric *P. spicatum*, *P. ovatum*, and *P. persicifolium* constitutes a new approach to clarifying the taxonomic statuses. The numeric analysis of color, providing colorimetric variables, together with the detailed description of the metabolic profiles of populations with different flower colors, constitute a unique chemical fingerprint that identifies species and subspecies with clear markers. This study is the most complete metabolic research on genus *Phyteuma*, since we identified and quantified 44 phenolic compounds using HPLC-MS, comprising 14 phenolic acids, 23 flavonols and flavones, and, for the first time in the genus, 7 anthocyanins involved in flower color variability. This approach contributes to clarifying the differences between species, which is particularly relevant in taxonomic complexes such as the present, where morphology fails to clearly differentiate taxa at specific and intraspecific levels.

## 1. Introduction

Genus *Phyteuma* (Campanulaceae) comprises 24 species distributed in Europe and Western Asia [1]. They are perennial herbs with linear to ovate leaves and solitary capitate to spicate inflorescences [2]. Their characteristic flowers have corollas that are deeply lobed at the base and fused at their tips, and their color varies between blue, violet, and white [1].

For genus *Phyteuma*, seven species have been reported in Slovenia: *P. orbiculare*, *P. ovatum*, *P. pseudorbiculare*, *P. scheuchzeri*, *P. sieberi*, *P. spicatum* and *P. persicifolium*. They are mainly identified by the inflorescence shape (cylindrical to ovoid), flower color and leaf size and shape [1,3]. In the genus, the taxonomic delimitation of some species is difficult since historical descriptions are not detailed and lead to misinterpretations [4]. Such is the case of *P. spicatum* and *P. ovatum*, where no consistent morphologic difference is observed except for flower color—white in *P. spicatum* and blue in *P. ovatum* [5]. However, in *P. spicatum*, two subspecies have been recognized, *P. spicatum* ssp. *spicatum* and *P. spicatum* ssp. *coeruleum*. They differ only in flower color—white in *P. spicatum* ssp. *spicatum* and light blue in *P. spicatum* ssp. *coeruleum* [1].

Flower color has historically been a relevant feature for identification of plant species in different genera. In genus *Phyteuma*, the use of flower color as the only taxonomically relevant feature for the identification of species and subspecies leads to many identification problems, not only because color cannot be observed in dry herbarium specimens, but also because, in nature, transitional colors can be found, and the subjective description of these variations can lead to misidentifications. These transitions in flower color could be a result of hybridization between co-occurring taxa, such as *P. spicatum*, *P. ovatum* and *P. persicifolium*, or even between the abovementioned subspecies of *P. spicatum* [4].

Although color differentiation has been mainly qualitative in traditional taxonomy, either only descriptive or using colorimetric cards, the subjective error of those kinds of color determinations is far from being objective and comparable. Therefore, quantitative numeric methods have been incorporated to clearly establish the differences between varieties, especially in species where a wide variety of colors are found [6,7,8,9]. Besides, the identification and quantification of anthocyanins can identify which of them are responsible for color variations and explain differences even between cultivars [10,11]. No such analyses have been carried out in *Phyteuma* yet.

Besides anthocyanins, other phenolic compounds, such as phenolic acids, flavones and flavonols, are involved in flower color formation, not only because they share precursors along their synthesis pathway [12] but also because they could be cofactors in the co-pigmentation effect [13,14,15]. The synthesis of anthocyanins and other phenolic compounds is related through the phenylpropanoid/flavonoid pathway, so changes in their contents could be related. Secondary metabolite profiles, mainly phenolic compounds, can also constitute chemical footprints by themselves that help with the identification of species [10,16]. This metabolomic approach has already proved to be a useful chemotaxonomic marker in different families [17,18] and also in Campanulaceae, specifically in the *Campanula pyramidalis* taxonomic complex [19].

This metabolic characterization has been useful in some species of *Phyteuma* before. Particularly, very specific saponins—phyteumosides—have been identified in the genus [20], which differentiates it from other related genera as these compounds are scarce in the Campanulaceae family [21]. Since some phenolic compounds show antioxidative properties and are involved in health-promoting effects, some of them have already been described in a few *Phyteuma* flowers and leaf rosettes since they have been traditionally consumed in salads [22]. In those analyses, the phenolic profiles do not differ between flowers of *P. spicatum, P. ovatum* and *P. orbiculare*, but are clearly different in *P. hemisphaericum*. In leaves, chemical profiles of *P. ovatum, P. orbiculare*, and *P. hemisphaericum* are similar, but those of *P. spicatum* show clear differences.

Since morphology by itself shows some difficulties in identifying clear groups that absorb and classify natural populations’ variability adequately, there is an increasing need to find new approaches and techniques that help clarify taxonomic relationships between taxonomic entities. We consider that a numerical analysis of color would clarify the differences in flower color and that the profile of secondary metabolites could constitute a unique fingerprint that identifies species and subspecies. The aim of this work is to (1) numerically describe color variation in flowers of the *P. spicatum–P. ovatum* taxonomic complex and the sympatric *P. persicifolium*, (2) identify which anthocyanins determine the color variation, (3) screen other phenolic compounds (phenolic acids, flavones, and flavonols) and quantify their content, and (4) analyze the variation of phenolic compounds at a specific and infraspecific level, along with color variation.

## 2. Results

### 2.1. Analysis of Color

#### 2.1.1. Color Variation in Natural Populations

**Figure 1:**
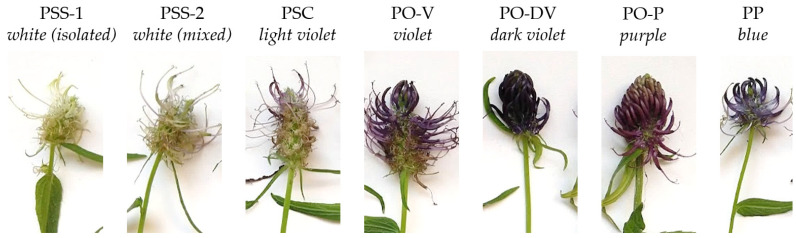
Color variation in *Phyteuma* species from Pohorje, Slovenia. PSS-1: *P. spicatum* ssp. *spicatum* populations growing alone; PSS-2: *P. spicatum* ssp. *spicatum* growing in sympatry with *P. spicatum* ssp. *caeruleum*; PSC: *P. spicatum* ssp. *caeruleum*; PO-V: *P. ovatum* populations with violet flowers; PO-DV: *P. ovatum* populations with dark violet flowers; PO-P: *P. ovatum* populations with purple flowers; PP: *P. persicifolium*.

In the field, natural populations showed a wide variation in flower color (Figure 1):

- *P. spicatum* ssp. *spicatum* (abbreviated further as PSS) showed white-greenish flowers. Some populations were growing alone (PSS-1), while others were growing in sympatry with *P. spicatum* ssp. *caeruleum* (PSS-2);
- *P. spicatum* ssp. *caeruleum* (PSC) showed white flowers with a very light violet tone;
- *P. ovatum* (PO) shows a typical violet color, although a wider variety of colors were observed on the field. The individuals with violet flowers were separated into two subgroups: one with typical violet flowers (PO-V) and the other with dark violet flowers (PO-DV). Some individuals showed purple flowers with a high presence of reddish tones (PO-P);
- *P. persicifolium* (PP) showed typical blue color.

#### 2.1.2. Numeric Analysis of Color

**Figure 2:**
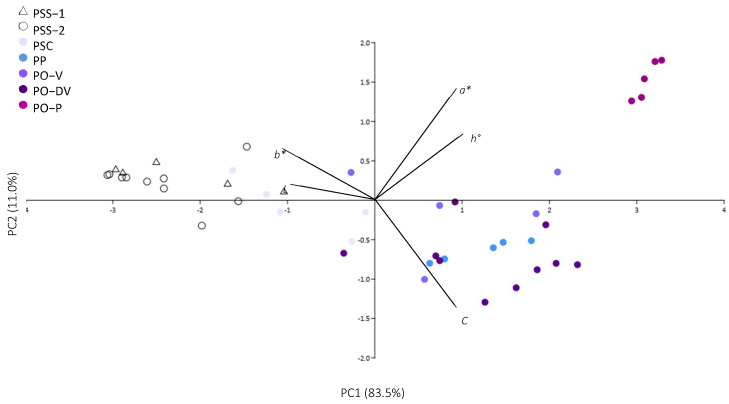
Principal component analysis of colorimetric variables in *Phyteuma*. PSS-1: *P. spicatum* ssp. *spicatum* populations growing alone; PSS-2: *P. spicatum* ssp. *spicatum* growing in sympatry with *P. spicatum* ssp. *caeruleum*; PSC: *P. spicatum* ssp. *caeruleum*; PO-V: *P. ovatum* populations with violet flowers; PO-DV: *P. ovatum* populations with dark violet flowers; PO-P: *P. ovatum* populations with purple flowers; PP: *P. persicifolium*.

Colorimetric evaluation of flowers from *Phyteuma* species (Table 1, Figure 2) supported the differences observed on the field (Figure 1). Regarding white-colored PSS, there was almost no difference in color tone between flowers from populations growing alone (PSS-1) and populations growing in sympatry with the other subspecies (PSS-2), except for color lightness, which was slightly higher in white flowers growing in isolated populations.

In the case of violet-colored flowers of PSC, PO-V, and PO-DV, the colorimetric analysis revealed marked differences between colors. With different tones of violet (from light to dark violet), lightness (L*) decreased and intensity (C*) increased, and the tones switched towards a higher presence of blue (more negative *b* values). Purple-colored flowers, however, showed a significantly higher proportion of red (highest positive *a* values) than violet flowers, while lightness and intensity did not differ so markedly.

Last, blue-colored PP showed very similar colorimetric variables as violet-colored PO-V and PO-DV, and are distant from both purple-colored PO-P and white PSS. However, it showed a significantly higher presence of blue (more negative *b* values) than light violet PSC.

### 2.2. Phenolic Compounds Identification and Content

#### 2.2.1. Anthocyanins

In *Phyteuma* species, seven anthocyanins were identified by their specific spectral data: delphinidin-3-rutinoside, cyanidin-3-rutinoside, peonidin-3-glucoside and derivatives of delphinidin rutinoside, petunidin-3-rutinoside, pelargonidin-3-rutinoside and delphinidin hexoside (Table 2). All of them are glycosylated, and some of them also show additional unidentified chemical groups, here identified as derivatives.

Anthocyanin content varied greatly among *Phyteuma* populations (Table 3). White-colored flowers of PSS showed no anthocyanin content, except for slight traces in PSS-2, which suggest the presence of peonidin-3-glucoside (Po3G) but not in a significant amount. Blue and purple-colored flowers showed the highest diversity of anthocyanins, followed by violet flowers. In light violet-colored flowers of PSC, delphinidin-3-rutinoside (D3R), cyanidin-3-rutinoside (C3R) and Po3G were detected, both in low content (5.8 and 2.2 mg/100 g FW, respectively). In violet-colored species (PO-V and PO-DV), D3R and C3R were the main anthocyanins, along with a petunidin rutinoside derivative (PtRd). However, the contents of these anthocyanins differed significantly between populations. In violet flowers, C3R showed the highest contents, followed by D3R, while in dark violet flowers, the relationship between the content of these anthocyanins was inversed. The content of PtRd in dark violet flowers was higher than in violet ones. The purple-colored flowers (PO-P) also contained D3R and C3R, although the contents differed significantly since D3R showed lower contents than violet-colored flowers, C3R showed the highest contents among all the compounds and populations (1511 mg/100 g FW). In purple flowers, PtRd was not detected as in violet flowers, but two other anthocyanins were detected: Po3G, in similar contents than in PSC, and a derivative of pelargonidin-3-rutinoside (PlRd) in relatively high contents.

Finally, blue-colored PP flowers showed a different anthocyanin pattern. In this case, C3R was the anthocyanin with the highest content, along with D3R and PlRd with low contents. Two anthocyanins were detected only in this species: A derivative of delphinidin rutinoside (DRd), in relatively high contents, and traces of a delphinidin hexoside derivative (DHd).

#### 2.2.2. Other Phenolic Compounds

Apart from anthocyanins, in the studied *Phyteuma* species, 35 other phenolic compounds were identified, comprising 14 phenolic acids and 23 flavonols and flavones (Table 4). They were identified based on the mass-to-charge ratios (*m*/*z*) and their fragmentation patterns. Among phenolic acids, there were mainly derivatives of chlorogenic (caffeoylquinic), *p*-coumaric and ferulic acids. Among flavonols, the main compounds were derivatives of quercetin, isorhamnetin and kaempferol, as well as thangenioside.

**Figure 3:**
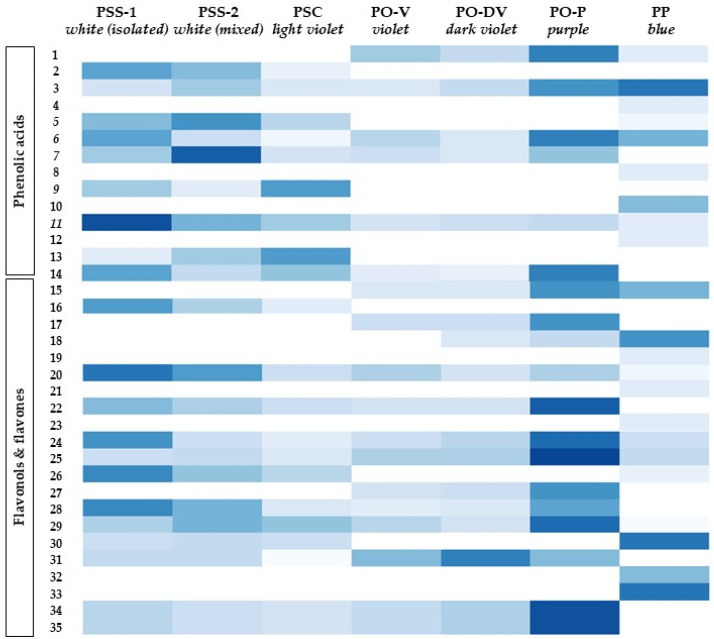
Distribution and content of phenolic compounds in *Phyteuma*. The color scale indicates the intensity of each compound amount between populations. Higher color intensity indicates higher amounts of each compound. PSS-1, *P. spicatum* ssp. *spicatum* populations growing alone; PSS-2, *P. spicatum* ssp. *spicatum* growing in sympatry with *P. spicatum* ssp. *caeruleum*; PSC, *P. spicatum* ssp. *caeruleum*; PO-V, *P. ovatum* populations with violet flowers; PO-DV, *P. ovatum* populations with dark violet flowers; PO-P, *P. ovatum* populations with purple flowers; PP, *P. persicifolium*.

*Variation among taxonomic entities.* The phenolic acids, flavones and flavonols profiles varied considerably among species and subspecies, regardless of flower color (Figure 3, Table S1). Some of them were restricted only to one species, such as compounds 2, 9, 13 and 18 in PS (PSS-1, PSS-2 and PSC) or compounds 15, 19 and 29 in PO (PO-V, PO-2 and PO-3). PP showed the most different phenolic profile of the species considered since almost 25% of all the phenolic compounds identified were restricted only to this species (compounds 4, 8, 10, 12, 21, 23, 25, 34 and 35).

Considering the intraspecific variation of PS, there was almost no difference either in the phenolic profiles or their contents between both subspecies (PSS and PSC), as well as between isolated (PSS-1) and mixed (PSS-2) populations of PSS.

Seven compounds were shared only between PS and PO (compounds 7, 14, 22, 28, 31, 34 and 35), while only three were between PO and PP (compounds 1, 15 and 18) and between PS and PP (compounds 5, 26 and 30). Although eight compounds were present in all the species (compounds 3, 6, 11, 20, 24, 25 and 29), their content was not always similar between the species. In some of them, the content was constant, such as in compounds 3 and 6, while in others they varied between species, sometimes with the highest contents in PS or PO (compounds 11, 20, 24, 25 and 29) and others in PP (compound 16).

*Variation among populations with different flower colors.* There seemed to be less variation in phenolic acids, flavones and flavonols’ profiles among flower colors (Figure 3, Table S1). On an overall view, blue and purple-colored flowers showed the most different phenolic profiles of all populations considered. Purple flowers (PO-P) showed a significantly higher content of compounds 1, 14, 15, 17, 22, 24, 25, 27, 28, 34 and 35 than violet flowers.

Although there was no difference in the phenolic profile or contents of individual phenolics between violet and dark violet flowers (PO-V and PO-DV), there was a different phenolic profile in light violet (PSC) flowers. Some compounds varied with violet intensity, either increasing (e.g., compounds 25, 31 and 34) or decreasing (e.g., compounds 11 and 14).

#### 2.2.3. Total Contents of Phenolic Compounds

**Figure 4:**
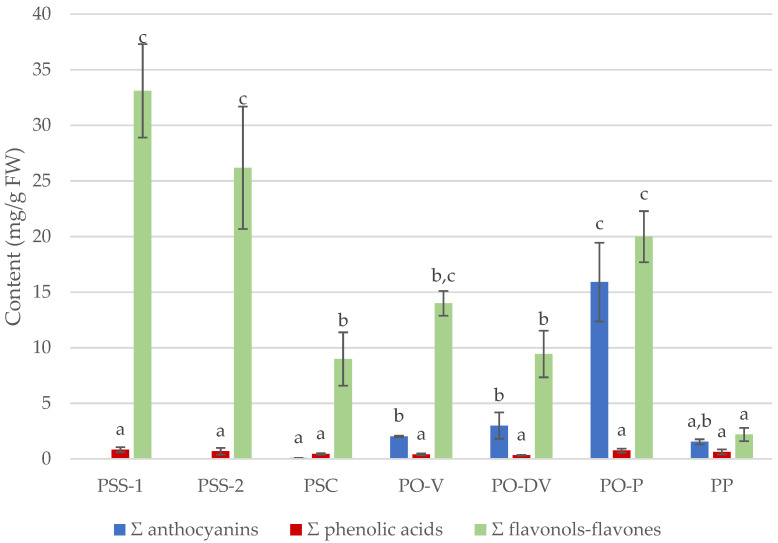
Total sum (Σ) of anthocyanin, phenolic acid and flavonol–flavone contents (mean ± SD, in mg/g FW) in different species of *Phyteuma*. Different letters indicate statistical differences between populations. PSS-1, *P. spicatum* ssp. *spicatum* populations growing alone; PSS-2, *P. spicatum* ssp. *spicatum* growing in sympatry with *P. spicatum* ssp. *caeruleum*; PP, *P. persicifolium*; PSC, *P. spicatum* ssp. *caeruleum*; PO-V, *P. ovatum* populations with violet flowers; PO-DV, *P. ovatum* populations with dark violet flowers; PO-P, *P. ovatum*, populations with purple flowers.

Although there was no difference in total values of phenolic acids among species or colors, significant differences were detected in total flavonols–flavones and anthocyanins contents among populations (Figure 4).

The lowest total flavonoid content was detected in blue-colored PP and purple-colored PO-P, and the highest in white-colored PSS. Regarding total anthocyanin content, purple-colored PO-P showed considerably highest values than any other population, followed by violet PO. Although there was no difference in any total contents between isolated and mixed populations of PSS (PSS-1 and PSS-2), significant differences were observed in total flavonoid content between the subspecies (PSS and PSC). PO showed intermediate values of total flavonoid content compared to PP and PS.

**Figure 5:**
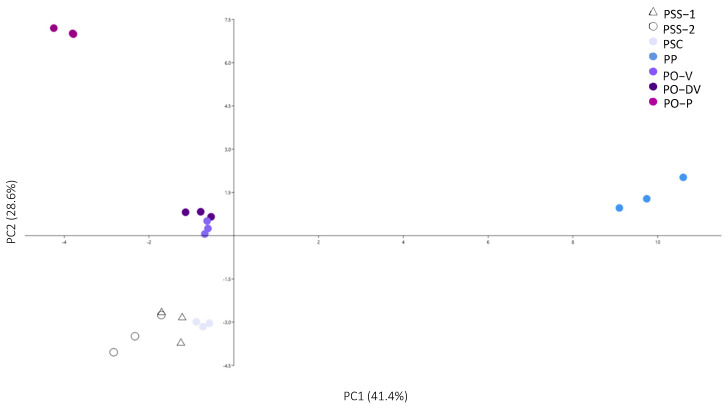
Principal component analysis of all phenolic compounds in *Phyteuma*. PSS-1, *P. spicatum* ssp. *spicatum* populations growing alone; PSS-2, *P. spicatum* ssp. *spicatum* growing in sympatry with *P. spicatum* ssp. *caeruleum*; PSC, *P. spicatum* ssp. *caeruleum*; PO-V, *P. ovatum* populations with violet flowers; PO-DV, *P. ovatum*, populations with dark violet flowers; PO-P, *P. ovatum* populations with purple flowers; PP, *P. persicifolium*.

Considering all phenolic groups (anthocyanins, phenolic acids and flavonols–flavones), the complete phenolic profile showed low intra-population variability (Figure 5). It clearly separated into 4 groups: (1) PP, (2) PO-3, (3) PO-V and PO-DV, and (4) PSS-1 and PSS-2. However, PO-V and PO-DV in group 3 were not so clearly separated, as well as PSS-1 and PSS-2 in group 4. The phenolic compounds that influenced the distribution along PC1 the most were phenolic compounds number 4, 15, 8, 10, 12, 18, 19, 21, 22, 23, 28, 29, 30, 32 and 33, and anthocyanins 4 and 7 (Table 2, Table 4 and Table S2). These compounds were mainly the ones that were exclusive to PP or whose amounts were very different in PP compared to the other populations. In PC2, the most important compounds were numbers 1, 3, 5, 15, 9, 17, 13, 25, 27, 34 and 35, and anthocyanins 2 and 6 (Table 2, Table 4 and Table S2). These compounds were absent in PO, exclusive to it, or whose amounts were very different in PO compared to the other populations.

## 3. Discussion

### 3.1. Numeric Analysis of Color

The numeric analysis of color with a colorimeter is a simple and accessible tool for an accurate assessment of color variation, and it was also useful for differentiating cultivars in *Lobelia* [11]. This type of analysis is therefore crucial not only for taxonomic purposes but also for flowering plant breeders, which can, in this way, ensure the proper characterization of the genetic material and use it for a further selection of new varieties. Our research on flower color in *Phyteuma* shows that numeric analysis of color is useful to separate white and purple-colored flowers but is not enough to clearly separate blue and violet tones by itself. However, the flower color could still be used to separate both species in combination with other morphological traits [3].

### 3.2. Phenolic Compounds Identification and Content

#### 3.2.1. Anthocyanins

The detailed analysis of anthocyanin composition in flowers has proven to be taxonomically relevant in *Campanula* sp., where blue-flowering phenotypes were identified based on their anthocyanin composition [23]. It was reported that leucodelphinidin and leucoanthocyanins are absent in the family [21], and they have not been found in our samples either, probably because they are intermediates in the anthocyanin synthesis pathway [12] and would be highly unstable. On the other hand, delphinidin has been identified as the major compound in blue-colored flowers of other species from the Campanulaceae family [24,25]. However, in blue-colored PP, the most abundant anthocyanin is cyanidin, while delphinidin mainly dominates in violet-colored PO.

The analysis of anthocyanins revealed that in violet-colored populations of PO-V and PO-DV, the anthocyanins are the same, but their contents differ, mainly increasing in PO-DV, which explains the increase in color intensity observed in the field, and also in the colorimetric analysis results. A similar effect has also been observed in *Paeonia* flowers, where variation in color intensity correlated with an increase in anthocyanin concentration, as well as other metabolic and hormone-mediated signaling pathways involving differential expression of anthocyanin-related genes [26]. These results suggest that the variation in the intensity of the violet color of PO-V and PO-DV by itself does not justify an establishment of a different subspecies of PO.

The purple color of PO-P flowers is a result of a major change in the composition and content of anthocyanins. The final color is then explained by the increase in Po3G and PlRd, which adds reddish tones, and the decrease of D3G, which diminishes the blue tones in the flowers [27]. The causes and genetic stability of this shift in the enzyme activity of anthocyanidin synthase remain to be understood.

Genus *Phyteuma* is well known for its high hybridization in some groups of species [2,4]. Regarding this, the populations of PS and PO are also interesting cases since both species are at some point sympatric [1] and hybridization between them might be possible. Many taxonomic reports have suggested the interbreeding of PS and PO and the consequent presence of populations with intermediate flower colors [5,28,29]. Moreover, some populations were even identified as different subspecies [30], which was also suggested for PSC before [31]. Until further genetic studies of these populations are available, the anthocyanin profile could shed some light on this question. Color in PSC is explained by an increase in the content of Po3G, which is absent in isolated populations of PS (PSS-1) and detected (although in traces) in populations of PS growing in sympatry with PSC (PSS-2). The anthocyanin profile suggests that PSC could be a result of the hybridization between PSS, with no anthocyanins, and PO, since they show a high content of D3R and the presence of Po3G, while in PP content of D3R are lower, and Po3G is absent. It was suggested that PO may be a restricted variant of PS limited to some geographical regions and high altitudes [30,32]. Considering its contrasting anthocyanin profiles, this would be possible if a genomic silencing of anthocyanin-related genes in PS would be removed in PO, allowing the expression of color-related compounds [33,34].

#### 3.2.2. Other Phenolic Compounds

In the Campanuloideae subfamily, the most abundant phenolic compounds are the der.s of caffeic acid, mainly chlorogenic (esters with quinic acid) and *p*-coumaric acids [21]. Indeed, in some species of the genus *Phyteuma*, the most abundant phenolic compounds were identified as thansgenioside, chlorogenic acid, and luteolin derivatives [22]. Our results coincide with these studies and contribute to a more detailed phenolic profile of the genus as well since high amounts of quercetin and isorhamnetin derivatives were identified and quantified as well. Higher amounts of quercetin are relevant in populations in which Po3G and C3R were detected since precursors of quercetin are also involved in the synthesis of both these compounds [12].

Considering the taxonomic relevance of the whole phenolic profile, PP shows a significantly different profile from all the other species, showing marked differences not only in the composition of the compounds but also in their content. This could be related to their phylogenetic distance [4] and also to their significant morphologic differences [3]. On the other hand, PS also show a slightly different phenolic profile from PO, but not as high as with PP, which is consistent with their phylogenetic proximity [4] and their morphologic [3] and metabolic similarity [22]. Although in some studies PS and PO are defined as different species [1,3], their delimitation is not clear and some studies suggest that PO is a subspecies of PS, as well as PSC [5].

The numeric and metabolic analysis of the flower color of PO-P is also an interesting case. Although it is identified as PO by traditional taxonomic keys [3], our results indicate that this population clearly differs from typical PO populations (PO-V and PO-DV). Therefore, the identification of populations with purple flowers as a different subspecies of PO seems possible.

In the case of PSS, populations with white flowers show the same phenolic profile, regardless if they grow isolated or in sympatry with PSC, which suggests that the establishment of two different subspecies is consistent in this species. The marked difference in the phenolic profile of PP from all the other species suggests that there is probably little hybridization between the mentioned species and that the color changes observed in PSC are more likely to be linked to the interbreeding of PS and PO, which has been suggested before [5], and not with PP, despite the fact that they are sympatric at some locations [1].

Since anthocyanins, phenolic acids, and flavonoid synthesis are connected through the phenylpropanoid metabolic pathway [35], related changes in their contents could be expected. In that study, it was described that the most frequent phenolic compounds in all flowers are *m*-coumaric acid, quercitrin, quercetin, and kaempferol, while *p*-hydroxybenzoic, caffeic, chlorogenic, and ferulic acid are much less frequent. Our results in the genus *Phyteuma* describe a much more diverse palette of phenolic compounds, mainly der.s from chlorogenic (caffeoylquinic), *p*-coumaric and ferulic acids, as well as derivatives of quercetin, isorhamnetin, and kaempferol. Interestingly, the profiles include many of the compounds described as less frequent, such as *p*-hydroxybenzoic acid and chlorogenic acid, which could additionally represent a chemotaxonomic marker of the genus *Phyteuma* at suprageneric levels.

Besides the phenolic compounds that are present regardless of species and color, it has been described that some phenolic profiles could be associated with specific colors, such as the presence of *p*-coumaric acid, vanillic and syringic acids, and myricetin in violet flowers, and the absence of them in blue flowers [36]. At an infrageneric level of genus *Phyteuma*, from the abovementioned metabolites, only *p*-coumaric acid has been detected, but in both violet and blue flowers, although in blue flowers, the amount is significantly lower. However, considering the whole metabolic profile, the association between metabolites and flower color seems to be more species-specific rather than color-specific.

Phenolic acids, flavones, and flavonols are also related to the pigmentation of flowers by their co-pigmentation effect, increasing color intensity through glycosylation and acylation of anthocyanins [13,14]. Among them, flavonols such as catechin [15] and phenolic acids such as chlorogenic and *p*-coumaric acids [11,37] are more frequently associated with this effect. The analysis of the complete profile of phenolic compounds along with anthocyanins is, therefore, crucial. In fact, our results indicate that anthocyanins are indeed glycosylated and modified with other unidentified chemical groups. An increase in the amounts of flavonols along with color intensity in PO was found, which suggests that an additional co-pigmentation effect could also be involved in the final color of *Phyteuma* flowers. Dark violet and purple-colored flowers of PO-DV and PO-P show the highest contents of most phenolic compounds. Therefore, their darker color could be a result not only of the anthocyanin composition and content but also of the highest contents of phenolic compounds that enhance their color.

## 4. Materials and Methods

### 4.1. Plant Material

Five individuals per population of *P. spicatum*, *P. ovatum*, and *P. persicifolium* were collected on 10 July 2021 in the Pohorje region, Slovenia (46°30′47.0″ N 15°11′12.0″ E). All samples were identified following Slovenian taxonomic keys for the genus [3]. Seven different populations with visually different flower colors were detected and sampled separately. After identification and color evaluation (see Section 4.2), samples were frozen with liquid nitrogen and stored at −20 °C until further analysis.

### 4.2. Color Evaluation

Flower color was evaluated on the inflorescences of each population using a Konica Minolta CR-10 Chroma portable colorimeter (Tokyo, Japan), which works with the CIELAB standard [38]. Colors are described with five parameters: L* (lightness on a 0–100 dark-bright scale), *h°* (hue angle: 0°–90° is red towards yellow, 90°–180° is yellow towards green, 180°–270° is green towards blue and 270°–360° is blue towards red), *a* and *b* (−60 to 60 from green to red and from blue to yellow) and C* (increasing intensity of color). For each population, five measures were made in the central portion of the inflorescence.

### 4.3. Extraction of Phenolic Compounds

Extractions were performed as described by [38]. For each population, 0.3 g of fresh squashed flowers were immersed in 2 mL of methanolic solution (70% Methanol, 27% bi-distilled water and 3% formic acid). Three repetitions per population were prepared. All samples were placed in an ultrasonic bath for 30 min and then centrifuged at 8000× *g* and 4 °C for 7 min (5810 R; Eppendorf, Hamburg, Germany). The supernatants were then filtered into vials through 0.2 µm Chromafil^®^ AO-20/25 (Macherey-Nagel, Düren, Germany) polyamide filters.

### 4.4. Analysis with HPLC-MS

Phenolic acid, flavonoid, and anthocyanin profiles were obtained following the method described by [38]. A Dionex HPLC system (Thermo Fisher Scientific, Waltham, MA, USA) with a diode array detector at 280 nm for phenolic acids, 350 nm for flavonols-flavones, and 530 nm for anthocyanins was used. A Phenomenex HPLC column C_18_ (150 × 4.6 mm, Gemini 3 μm) was heated at 25 °C. Phenolic acids, flavonols, and flavones were identified and quantified by comparing their UV-Vis spectra and retention times with standards and also confirmed with a mass spectrometer (Thermo Fisher Scientific, LCQ Deca XP MAX) with an electrospray interface (ESI) operating in negative ion mode, while anthocyanins were scanned in positive ion mode. Full scan data-dependent MS^n^ scanning from *m*/*z* 115 to 2000 was performed. All conditions on the mass spectrometer were reported before [38].

All compounds were identified based on literature, both anthocyanins [39] and other phenolic compounds [22,40,41,42,43,44,45,46,47]. They were quantified based on the corresponding external standards. If it was not available, they were calculated on a related standard: compound 10 on chlorogenic acid, comp. 8, 9 and 12 on ferulic acid, comp. 23, 24, 26, 27, 33, 34 and 35 on isorhamnetin-glucoside, comp. 19 on kaempferol-glucoside, comp. 29 on kaempferol-rutinoside, comp. 16 and 20 on luteolin-glucoside, comp. 1, 2, 3, 6, 11, 13, 14 and 22 on *p*-coumaric acid, comp. 17, 25, 28, 30, 31 and 35 on quercetin-glucoside, and comp. 32 on quercetin-rutinoside. Among anthocyanins, compounds 1, 4 and 7 were calculated on delphinidin-3-*O*-glucoside chloride standard, comp. 2 on cyanidin 3-*O*-galactoside chloride, comp. 3 on peonidin chloride, comp. 5 on petunidin chloride, and comp. 6 on pelargonidin chloride. All contents are expressed as mg/100 g of fresh weight (FW).

### 4.5. Chemicals

HPLC-grade methanol and formic acid for the extraction of the phenolics were purchased from Sigma-Aldrich (Steinheim, Germany). For the mobile phases, we used HPLC–MS grade acetonitrile and formic acid from Fluka Chemie (Buch, Switzerland). The following standards were used for the quantification of phenolic compounds: Fluka Chemie (Buch, Switzerland): quercetin-3-glucoside (≥90%), *p*-coumaric acid (≥98.0%), kaempferol-3-rutinoside (≥95.0%), and kaempferol-3-glucoside (≥90.0%), delphinidin-3-*O*-glucoside chloride (≥95.0%), peonidin chloride (≥97%), and pelargonidin chloride (≥97%); Sigma-Aldrich: quercetin-3-rutinoside (≥90.0%), ferulic acid (≥99.0%), luteolin 7-*O*-*β*-D-glucoside (≥98%), 3-caffeoylquinic acid (≥95%) and 5-caffeoylquinic acid (≥95.0%); Extrasynthese (Genay, France): isorhamnetin-3-glucoside (≥95.0%), cyanidin 3-*O*-galactoside chloride (≥97%) and petunidin chloride (≥95%). The water for phenolic compounds extraction and mobile phases was double distilled and purified with a Mili-Q Millipore system (Merck Millipore, Billerica, MA, USA).

### 4.6. Statistical Analysis

For colorimetric parameters, analysis of variance (ANOVA) was performed using R 4.0.3, along with the multiple comparison Duncan test for statistical significance (*p* ≤ 0.05) between color groups of each compound. For concentrations of phenolic acids, flavonols-flavones, and anthocyanins, non-parametric analysis of variance (MANOVA) was performed, with the Kruskal–Wallis non-parametric test for statistical significance (*p* ≤ 0.05) between groups for each parameter. Two Principal component analyses (PCA) were conducted using R, one based on the 5 colorimetric variables and another with all the 44 phenolic compounds of each population on the correlation matrix.

## 5. Conclusions

In taxonomic complexes, where the delimitation of species and subspecies is sometimes unclear and does not reflect natural variability adequately, new tools are necessary to address the problem. In the genus *Phyteuma*, the numeric and chemical analysis of flower color constitutes a useful tool that helps to clarify taxonomic identifications at an infrageneric level. The numeric analysis of color included 5 parameters that clearly identified the flower color of each population. The metabolic profile of phenolic compounds, including 7 anthocyanins, 14 phenolic acids, and 23 flavonols–flavones, also constituted a solid chemotaxonomic marker to differentiate species and subspecies.

Both analyses clearly differentiate PO-P and PS, which strongly suggest that they are different taxonomic entities, confirming the status of PS as a different species and suggesting that populations with purple flowers could constitute a different subspecies, which should be complemented with intense morphologic and genetic studies. The analysis of color does not differentiate both PS subspecies by itself, as well as between populations of PSS growing alone or in sympatry with PSC, which do not support the differentiation of two subspecies, at least not solely on flower color. The observed variation in color that led to the establishment of PSC could be a result of the hybridization of PSS with PO and not with PP since their metabolic profile shows similarities between them and not with the latter.

The numeric and chemical analysis of color does not separate different tones of violet PO populations, confirming the identification of PO as a species with violet flowers, with some variation in color tones, although not enough to establish different subspecies. Numeric analysis of color does not differentiate violet-colored PO and blue-colored PP, but the metabolic profile clearly does, supporting their separate taxonomic entities.

In the future, wider research including all *Phyteuma* species from the whole geographical distribution would be useful to prove the consistency of these results at both generic and infrageneric levels and to better understand the metabolic pathways that determine the synthesis of anthocyanins and other phenolic compounds, which determine their flower metabolism and visual properties.

## Figures and Tables

**Table 1:** Colorimetric variables (mean ± SD) for different populations of *Phyteuma*, indicating *L* (lightness), *h°* (hue angle), *a* and *b* green–red and blue–yellow scale) and C* (intensity). Different letters indicate statistical differences between populations.

Group	Flower Color	L*	*a*	*b*	C*	*h°*
PSS-1	white	58.3 ± 5.3 ^e^	−0.7 ± 1.3 ^a^	−34.2 ± 2.7 ^c^	24.2 ± 2.7 ^d^	268.5 ± 3.0 ^c^
PSS-2	white	51.4 ± 2.9 ^d^	−0.5 ± 1.7 ^a^	−33.4 ± 4.9 ^c^	23.4 ± 4.9 ^d^	269.5 ± 3.9 ^c^
PSC	light violet	45.8 ± 2.2 ^c^	1.6 ± 0.8 ^b^	−41.8 ± 4.9 ^b^	31.7 ± 4.9 ^c^	275.6 ± 4.1 ^c^
PO-V	violet	37.2 ± 3.3 ^b^	4.2 ± 1.7 ^c^	−51.0 ± 4.5 ^a^	39.6 ± 4.5 ^b^	299.4 ± 20.2 ^b^
PO-DV	dark violet	29.9 ± 6.4 ^a^	2.6 ± 1.3 ^b^	−53.5 ± 4.5 ^a^	42.7 ± 4.2 ^a,b^	298.0 ± 15.8 ^>b^
PO-P	purple	24.9 ± 2.9 ^a^	10.9 ± 0.9 ^d^	−55.8 ± 0.6 ^a^	38.3 ± 0.7 ^b^	339.1 ± 4.1 ^a^
PP	blue	42.2 ± 1.2 ^b,c^	3.0 ± 0.4 ^b,c^	−55.3 ± 1.8 ^a^	44.3 ± 1.3 ^a^	304.8 ± 13.4 ^b^

PSS-1: *P. spicatum* ssp. *spicatum* populations growing alone; PSS-2: *P. spicatum* ssp. *spicatum* growing in sympatry with *P. spicatum* ssp. *caeruleum*; PSC: *P. spicatum* ssp. *caeruleum*; PO-V: *P. ovatum* populations with violet flowers; PO-DV: *P. ovatum* populations with dark violet flowers; PO-P: *P. ovatum* populations with purple flowers; PP: *P. persicifolium*.

**Table 2:** Tentative identification of anthocyanins from Slovenian populations of *Phyteuma*, indicating molecular mass-to-charge ratio (*m*/*z*) and relative intensity between brackets.

Peak	RT (min)	ʎ_max_	[M + H]^+^ (*m*/*z*)	MS^2^ (*m*/*z*)	MS^3^ (*m*/*z*)	Tentative Identification
1	8.9	527	611	303(100), 465(16)		Delphinidin-3-rutinoside
2	10.2	517	595	287(100), 449(19)		Cyanidin-3-rutinoside
3	12.1	528	463	301(100)		Peonidin-3-glucoside
4	14.7	535	1175.6	867(100), 611(44), 465(5)	[465] 303(100); [611] 303(100), 465(17)	Delphinidin rutinoside der.
5	16.7	523, 533	727	317(100), 479(71)		Petunidin-3-rutinoside der.
6	18.6	530, 523	787.5	479(100), 299(29)	[479] 299(100); [299] 271(100), 255(24), 243(13)	Pelargonidin-3-rutinoside der.
7	22.6	542	551	303(100)		Delphinidin hexoside der.

Rt, retention time; [M + H]^+^, pseudo-molecular ion identified in positive ion mode; MS^2^, MS^3^, further fragmentations; numbers in [] are the parent ions; der., derivative.

**Table 3:** Anthocyanin content (mean ± SD, in mg/g FW) in *Phyteuma*. Different letters mean statistical differences between populations for each anthocyanin.

Population	Color	D3R	DRd	DHd	C3R	Po3G	PtRd	PlRd
PSS-1	white	-	-	-	-	-	-	-
PSS-2	white	-	-	-	-	traces	-	-
PSC	light violet	5.8 ± 1.6 ^a^	-	-	1.9 ± 0.6 ^a^	2.2 ± 0.9 ^a^	-	-
PO-V	violet	72.8 ± 41.7 ^b^	-	-	128.3 ± 52.5 ^a^	-	1.4 ± 0.7 ^a^	-
PO-DV	dark-violet	180.7 ± 80.1 ^a^	-	-	106.6 ± 58.6 ^a^	-	12.2 ± 8.7 ^b^	-
PO-P	purple	58.5 ± 21.3 ^b^	-	-	1511.3 ± 42.3 ^b^	1.2 ± 0.1 ^a^	-	19.3 ± 1.2 ^b^
PP	blue	1.4 ± 0.1 ^c^	13.7 ± 3.0 ^a^	traces	136.4 ± 33.8 ^a^	-	-	2.2 ± 1.9 ^a^

D3R, delphinidin-3-rutinoside; C3R, cyanidin-3-rutinoside; Po3G, peonidin-3-glucoside; DRd, delphinidin rutinoside derivative; PtR, petunidin-3-rutinoside derivative; PlRd, pelargonidin-3-rutinoside derivative; DHd, delphinidin hexoside derivative; PSS-1, *P. spicatum* ssp. *spicatum* populations growing alone; PSS-2, *P. spicatum* ssp. *spicatum* growing in sympatry with *P. spicatum* ssp. *caeruleum*; PSC, *P. spicatum* ssp. *caeruleum*; PO-V, *P. ovatum*, populations with violet flowers; PO-DV, *P. ovatum* populations with dark violet flowers; PO-P, *P. ovatum* populations with purple flowers; PP, *P. persicifolium*.

**Table 4:** Tentative identification of phenolic acids, flavones and flavonols from Slovenian populations of *Phyteuma*, indicating molecular mass-to-charge ratio (*m*/*z*) and relative intensity between brackets.

Peak	RT (min)	ʎ_max_	[M − H]^−^ (*m*/*z*)	MS^2^ (*m*/*z*)	MS^3^ (*m*/*z*)	MS^4^ (*m*/*z*)	Tentative Identification
	**Phenolic acids**						
1	8.03	279, 304	325	163(100)			*p*-Coumaric acid hexoside der.
2	8.5	261	299	137(100), 179(63)			*p*-Hydroxybenzoic acid hexose
3	9.04	312,279	325	163(100)			*p*-Coumaric acid der.
4	9.4	306	353	191(100), 179(46)			Neochlorogenic acid
5	9.9	322,297	353	191(100), 179(46)			Caffeoylquinic acid der.
6	12.5	310	337	163(100), 173(4), 119(4), 191(6)			Coumaroylquinic acid der. 1
7	13.0	318	353	191(100), 179(6)			Cryptoclorogenic acid
8	13.7	322,248	367	193(100), 134(5), 173(4)			Feruloylquinic acid der. 1
9	14.0	327,252	355	193(100), 175(30)			Ferulic acid hexoside
10	16.2	267, 311	677	502(100), 503(93), 323(13)	[502] 240(100), 191(74), 163(58), 173(31); [323] 179(100)	[163] 119(100), 163; [179] 135(100)	*p*-coumaric-caffeoylquinic acid der.
11	16.5	311	337	191(100), 163(6), 173(6)			Coumaroylquinic acid der. 2
12	18	273	705	531(100), 357(21)	[531] 357(100), 269(42), 313(16)	[357] 313(100), 193(13), 163(12)	Feruloylquinic acid der. 2
13	18.0	271	367	193(100), 173(8)			Ferulic acid der.
14	18.2	304	337	191(100), 163(6)			Coumaroylquinic acid der. 4
	**Flavonols & flavones**						
15	10.41	374	593	285(100), 284(29)			Kaempferol-3-*O*-rutinoside
16	15.6	377	755	593(100)	[593] 285(100)		Luteolin-7-rutinoside glucoside
17	17.3	350	755	593(100), 300(48), 301(23), 271(9)			Quercetin hexoside dirhamnoside
18	19.0	353	609	300(100), 301(27), 271(15), 179(4)			Quercetin-3-rutinoside 1
19	19.5	355	635	284(100), 285(30), 255(28)			Kaempferol der. 2
20	19.9	347	593	285(100)	[285] 285(100), 241(33), 175(24), 199(21), 217(20), 243(20)		Luteolin-7-rutinoside
21	20.1	351	609	301(100), 300(22), 179(2)			Quercetin-3-rutinoside 2
22	20.1	350	1338	497(100), 659(89)	[497] 261(100)		Tanghenioside VII
23	20.4	330,350	623	315(100), 300(51)			Isorhamnetin-3-*O*-rutinoside 1
24	20.6	351	623	314(100), 315(96), 299(32)			Isorhamnetin-3-*O*-rutinoside 2
25	21.07	351	463	301(100), 300(24), 179(2)			Quercetin hexoside
26	21.8	352	623	315(100), 300(17)			Isorhamnetin-3-*O*-rutinoside 3
27	21.9	352	623	315(100), 300(63), 271(5)			Isorhamnetin-3-*O*-rutinoside 4
28	22.0	344	653	611(100), 301(32), 300(29), 271(6)			Quercetin der. 1
29	22.3	345	608	300(100), 299(81), 285(25), 284(23)	[300] 285(100), 284(7); [299] 284(100)	[284] 284(100), 256(11)	Kaempferol dihexoside
30	22.6	353	549	505(100), 405(45), 345(11)	[505] 301(100), 300(22)		Quercetin malonyl-hexoside der. 1
31	22.6	353	1099	505(100), 549(25)			Quercetin malonyl-hexoside dimer
32	23.7	350	505	301(100), 300(63), 179(3)			Quercetin der. 2
33	24.6	353,355	563	531(100), 463(76)	[531] 463(100); [463] 301(100), 300(14), 179(1)		Isorhamnetin malonyl-glucoside
34	24.6	353,355	1127	519(100)	315(100), 300(12)		Isorhamnetin der. 1
35	24.6	353,355	519	314(100), 315(63)			Isorhamnetin der. 2

Rt, retention time; [M − H]^−^, pseudo-molecular ion identified in negative ion mode; MS^2^, MS^3^, MS^4^, further fragmentations; numbers in [] are parent ions; der., derivative.

## Data Availability

Most data presented in this study are available in Supplementary Material. The remaining data are available on request from the corresponding author.

## Supplementary Materials

The following supporting information can be downloaded at: https://www.mdpi.com/article/10.3390/plants11212894/s1, Table S1: Phenolic compounds content (mean ± SD, in mg/100 g of fresh weight) in selected populations of *Phyteuma*; Table S2: Loadings for the multivariate analysis (PCA) of metabolic compounds in *Phyteuma*; Table S3. Key to populations of *Phyteuma* included in this work; Figure S1: HPLC chromatograms of *Phyteuma* species at 530 nm. (A) *Ph. spicatum* ssp. *spicatum*; (B) *Ph. spicatum* ssp. *caeruleum*; (C) *Ph. ovatum*, populations with violet flowers; Figure S2: HPLC chromatograms of *Phyteuma* species at 530 nm (cont.). (A) *Ph. ovatum*, populations with dark violet flowers; (B) *Ph. ovatum*, populations with purple flowers; (C) *Ph. persicifolium*.
