# Supplementary material for: Color Variation and Secondary Metabolites’ Footprint in a Taxonomic Complex of Phyteuma sp. (Campanulaceae)
Source: Plants (Basel). 2022 Oct 28;11(21):2894. doi: 10.3390/plants11212894 (PMC9658285; doi:10.3390/plants11212894)
Supplement: Supplementary file 1 [file plants-11-02894-s001.zip › plants-1955098-Supplementary.pdf]

Table S1. Phenolic compounds content (mean ± SD, in mg/100 g of FW) in selected populations of *Phyteuma*. Different letters in the table indicate statistical significance ($p \leq 0.05$) in compound content between populations.

Peak	Compound	PSS-1	PSS-2	PSC	PO-V	PO-DV	PO-P	PP
	**Phenolic acids**							
1	*p*-Coumaric acid hexoside derivative	-	-	-	6.3 ± 0.3 b	4.6 ± 2.0 a,b	12.1 ± 2.5 c	2.1 ± 0.1 a
2	*p*-Hydroxybenzoic acid hexose	1.3 ± 1.0 a	1.1 ± 0.8 a	0.6 ± 0.3 a	-	-	-	-
3	*p*-Coumaric acid derivative	2.6 ± 0.8 a	3.8 ± 0.2 a	2.5 ± 0.6 a	2.5 ± 0.8 a	3.3 ± 0.2 a	5.6 ± 2.3 a	6.2 ± 2.2 a
4	Neochlorogenic acid	-	-	-	-	-	-	6.4 ± 0.4
5	Caffeoylquinic acid der.	8.8 ± 2.3 a	10.9 ± 7.3 a	6.7 ± 1.2 a	-	-	-	4.1 ± 0.1 a
6	Coumaroylquinic acid derivative 1	32.7 ± 12.9 a	20.7 ± 7.5 a	14.6 ± 0.5 a	23.9 ± 7.0 a	18.1 ± 0.4 a	38.3 ± 10.4 a	30.6 ± 18.0 a
7	Cryptoclorogenic acid	7.2 ± 0.8 a	15.1 ± 12.3 a	3.8 ± 0.5 a	4.1 ± 0.4 a	2.8 ± 0.4 a	7.6 ± 0.6 a	-
8	Feruloylquinic acid derivative 1	-	-	-	-	-	-	2.2 ± 0.3
9	Ferulic acid hexoside	1.5 ± 0.5 a	1.0 ± 0.1 a	1.9 ± 0.3 a	-	-	-	-
10	p-coumaric-caffeoylquinic acid derivative	-	-	-	-	-	-	8.8 ± 0.6
11	Coumaroylquinic acid derivative 2	21.3 ± 2.2 c	11.0 ± 0.2 b,c	7.4 ± 1.2 a,b	2.0 ± 0.2 a,b	2.7 ± 0.5 a,b	4.6 ± 0.8 a,b	0.3 ± 0.1 a
12	Feruloylquinic acid derivative 2	-	-	-	-	-	-	2.3 ± 1.5
13	Ferulicacid derivative	2.4 ± 1.1 a	3.3 ± 0.7 a	4.0 ± 0.7 a	-	-	-	-
14	Coumaroylquinic acid derivative 4	5.8 ± 2.2 b	3.0 ± 1.9 a,b	4.3 ± 1.2 a,b	1.9 ± 0.2 a	1.5 ± 0.3 a	6.9 ± 1.5 b	-
**Flavonols**								
15	Kaempferol-3-*O*-rutinoside	-	-	-	1.0 ± 0.6 a	0.5 ± 0.4 a	13.8 ± 2.5 b	10.2 ± 2.2 b
16	Luteolin 7-rutinoside glucoside	29.8 ± 21.5 a	17.9 ± 15.0 a	8.9 ± 6.5 a	-	-	-	-
17	Quercetin hexoside dirhamnoside	-	-	-	26.4 ± 3.9 a	25.0 ± 12.8 a	104.4 ± 14.0 b	-
18	Quercetin 3-rutinoside 1	-	-	-	1.1 ± 0.4 a	0.9 ± 0.2 a	2.5 ± 0.5 a	8.4 ± 0.9 b
19	Kaempferol derivative 2	-	-	-	-	-	-	2.8 ± 0.4
20	Luteolin 7-rutinoside	3386.1 ± 542.3 c	2392.3 ± 959.0 c	746.7 ± 252.1 b,c	1182.0 ± 75.0 b	678.1 ± 335.3 b,c	1207.4 ± 64.2 b,c	22.5 ± 8.1 a
21	Quercetin 3-rutinoside 2	-	-	-	-	-	-	1.8 ± 0.4
22	Tanghenioside VII	44.3 ± 12.3 b	33.9 ± 5.6 b,c	22.8 ± 4.2 a,b	19.5 ± 7.0 a	19.5 ± 5.9 a	82.1 ± 21.3 c	-
23	Isorhamnetin-3-*O*-rutinoside 1	-	-	-	-	-	-	3.0 ± 0.4
24	Isorhamnetin-3-*O*-rutinoside 2	16.1 ± 5.4 b	5.6 ± 2.8 a	3.6 ± 0.6 a	6.1 ± 1.1 a	8.0 ± 0.7 a	20.3 ± 5.5 b	5.6 ± 1.5 a
25	Quercetin hexoside	12.2 ± 7.1 a,b	13.6 ± 8.2 a,b	5.3 ± 0.5 a	17.9 ± 0.3 b	19.2 ± 9.1 b	56.4 ± 4.2 c	14.4 ± 2.0 a,b
26	Isorhamnetin-3-*O*-rutinoside 3	7.2 ± 1.4 a	5.1 ± 1.5 a	4.2 ± 1.2 a	-	-	-	2.1 ± 0.8 a
27	Isorhamnetin-3-*O*-rutinoside 4	-	-	-	4.3 ± 0.1 a	5.0 ± 0.1 a	17.6 ± 1.2 b	-
28	Quercetin derivative 1	9.5 ± 2.0 b	7.3 ± 4.5 a,b	4.0 ± 0.8 a	3.2 ± 0.3 a	3.7 ± 1.2 a	8.4 ± 1.4 b	-
29	Kaempferol dihexoside	42.3 ± 10.5 b	58.2 ± 4.4 b	51.0 ± 7.4 b	39.5 ± 7.0 b	30.3 ± 5.5 b,c	84.7 ± 9.9 c	12.7 ± 0.7 a
30	Quercetin malonyl-hexoside derivative	8.4 ± 4.8 a	12.5 ± 1.6 a	9.2 ± 3.5 a	-	-	-	63.4 ± 21.3 b
31	Quercetin malonyl-hexoside dimer	28.5 ± 11.2 a,b	29.7 ± 14.7 a,b	15.3 ± 5.4 a	39.2 ± 3.3 b	51.6 ± 16.2 b	39.0 ± 3.8 b	-
32	Quercetin derivative 2	-	-	-	-	-	-	6.4 ± 1.4
33	Isorhamnetin malonyl- glucoside	-	-	-	-	-	-	58.9 ± 16.3
34	Isorhamnetin derivative 1	48.8 ± 37.2 a,b	26.4 ± 11.3 a,b	17.1 ± 4.3 a	40.1 ± 8.4 a,b	69.1 ± 17.2 b	255.7 ± 85.2 c	-

35	Isorhamnetin derivative 2	26.9 ± 20.5 a	14.5 ± 6.2 a	9.4 ± 3.5 a	18.9 ± 4.0 a	32.5 ± 8.1 a	106.1 ± 15.4 c	-

PSS-1: *Ph. spicatum* ssp. *spicatum*, populations growing alone; PSS-2: *Ph. spicatum* ssp. *spicatum* growing in sympatry with *Ph. spicatum* ssp. *caeruleum*; PSC: *Ph. spicatum* ssp. *caeruleum*; PO-V: *Ph. ovatum*, populations with violet flowers; PO-DV: *Ph. ovatum*, populations with dark violet flowers; PO-P: *Ph. ovatum*, populations with purple flowers; PP: *Ph. persicifolium*.

Table S2. Loadings for the multivariate analysis (PCA) of metabolic compounds in *Phyteuma*.

Group	Peak No.	Compound ID	PC 1	PC 2
Phenolic acid	1	*p*-Coumaric acid hexoside derivative	-0.27104	0.91034
Phenolic acid	2	*p*-Hydroxybenzoic acid hexose	-0.30071	-0.65075
Phenolic acid	3	*p*-Coumaric acid derivative	0.40916	0.67752
Phenolic acid	4	Neochlorogenic acid	0.96202	0.16777
Phenolic acid	5	Chlorogenic acid	0.012282	-0.72262
Phenolic acid	6	Coumaroylquinic acid derivative 1	-0.014063	0.39072
Phenolic acid	7	Cryptoclorogenic acid	-0.53365	-0.1665
Phenolic acid	8	Feruloylquinic acid derivative 1	0.94616	0.15984
Phenolic acid	9	Ferulic acid hexoside	-0.2793	-0.75317
Phenolic acid	10	Caffeoylquinic acid derivative	0.95709	0.16486
Phenolic acid	11	Coumaroylquinic acid derivative 2	-0.45268	-0.55895
Phenolic acid	12	Feruloylquinic acid derivative 2	0.83569	0.16697
Phenolic acid	13	Coumaroylquinic acid derivative 3	-0.27131	-0.75961
Phenolic acid	14	Coumaroylquinic acid derivative 4	-0.69871	0.12154
Flavonoid	15	Kaempferol-3-*O*-rutinoside	0.35293	0.84087
Flavonoid	16	Luteolin-7-rutinoside glucoside	-0.28656	-0.64713
Flavonoid	17	Quercetin hexoside dirhamnoside	-0.4359	0.89083
Flavonoid	18	Quercetin-3-rutinoside 1	0.87483	0.44615
Flavonoid	19	Kaempferol derivative 2	0.94842	0.1607
Flavonoid	20	Luteolin-7-rutinoside	-0.56554	-0.41884
Flavonoid	21	Quercetin-3-rutinoside 2	0.95087	0.17403
Flavonoid	22	Tanghenioside VII	-0.73402	0.45823
Flavonoid	23	Isorhamnetin-3-*O*-rutinoside 1	0.96157	0.1674
Flavonoid	24	Isorhamnetin-3-*O*-rutinoside 2	-0.47106	0.55442
Flavonoid	25	Quercetin hexoside	-0.34704	0.88607
Flavonoid	26	Isorhamnetin-3-*O*-rutinoside 3	-0.15807	-0.60507
Flavonoid	27	Isorhamnetin-3-*O*-rutinoside 4	-0.43531	0.89168
Flavonoid	28	Quercetin derivative 1	-0.76714	0.016477
Flavonoid	29	Kaempferol dihexoside	-0.74083	0.31451
Flavonoid	30	Quercetin malonyl-hexoside derivative 1	0.93298	-0.002287
Flavonoid	31	Quercetin malonyl-hexoside dimer	-0.68697	0.21176
Flavonoid	32	Quercetin derivative 2	0.9352	0.15612
Flavonoid	33	Isorhamnetin malonyl-glucoside	0.94774	0.17415
Flavonoid	34	Isorhamnetin derivative 1	-0.55206	0.78542
Flavonoid	35	Isorhamnetin derivative 2	-0.56797	0.75587
Anthocyanin	1	Delphinidin-3-rutinoside	-0.22487	0.35098
Anthocyanin	2	Cyanidin-3-rutinoside	-0.3231	0.88887
Anthocyanin	3	Peonidin-3-glucoside	-0.29987	0.051944
Anthocyanin	4	Delphinidin rutinoside derivative	0.96074	0.17255
Anthocyanin	5	Petunidin-3-rutinoside derivative	-0.089675	0.088361
Anthocyanin	6	Pelargonidin-3-rutinoside derivative	-0.27085	0.84297
Anthocyanin	7	Delphinidin hexoside derivative	0.93093	0.15472

Figure S1. HPLC chromatograms of *Phyteuma* species at 530 nm. (A) *Ph. spicatum* ssp. *Spicatum*; (B) *Ph. spicatum* ssp. *caeruleum*; (C) *Ph. ovatum*, populations with violet flowers.

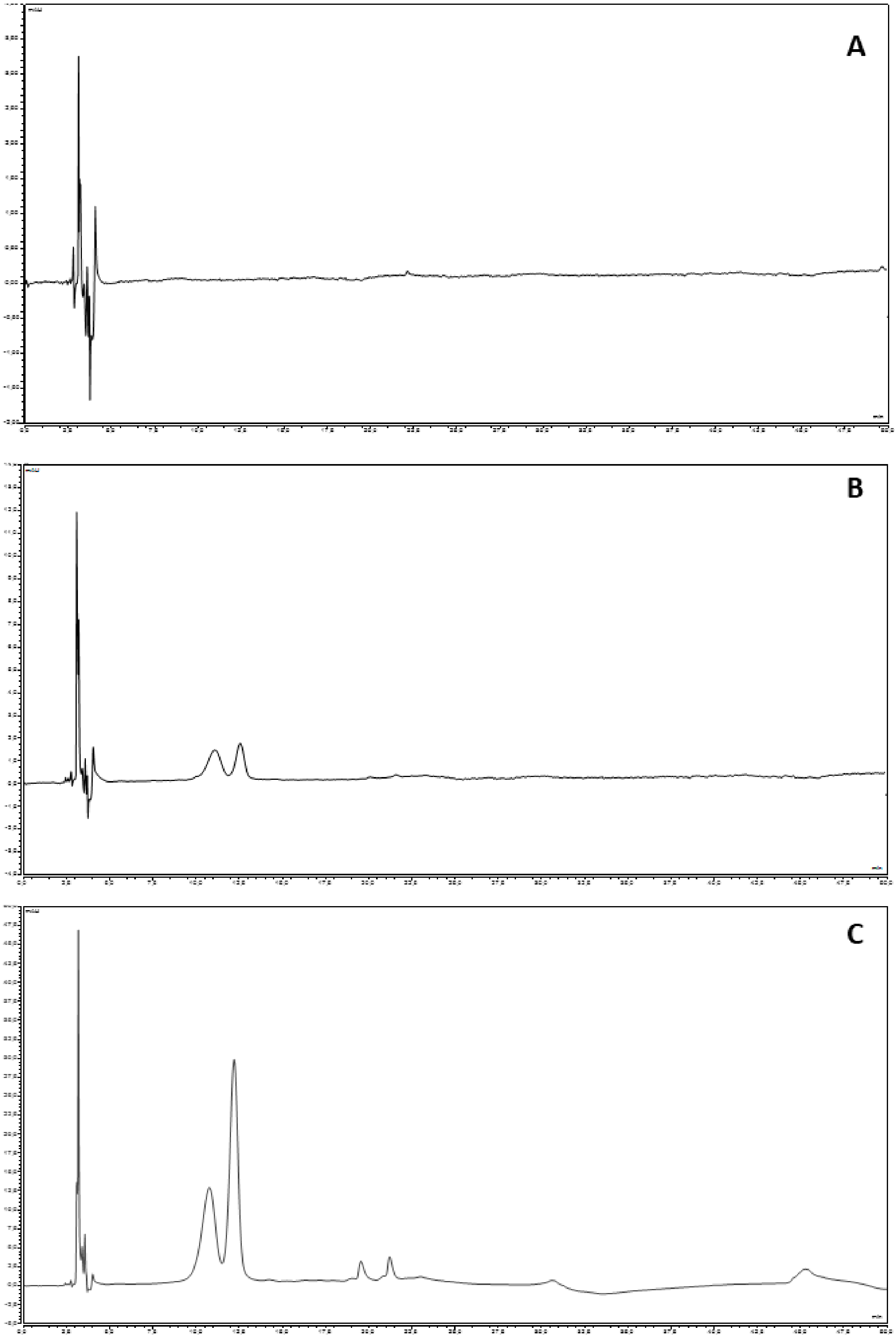

Figure S2. HPLC chromatograms of *Phyteuma* species at 530 nm (cont.). (A) *Ph. ovatum*, populations with dark violet flowers; (B) *Ph. ovatum*, populations with purple flowers; (C) *Ph. persicifolium*.

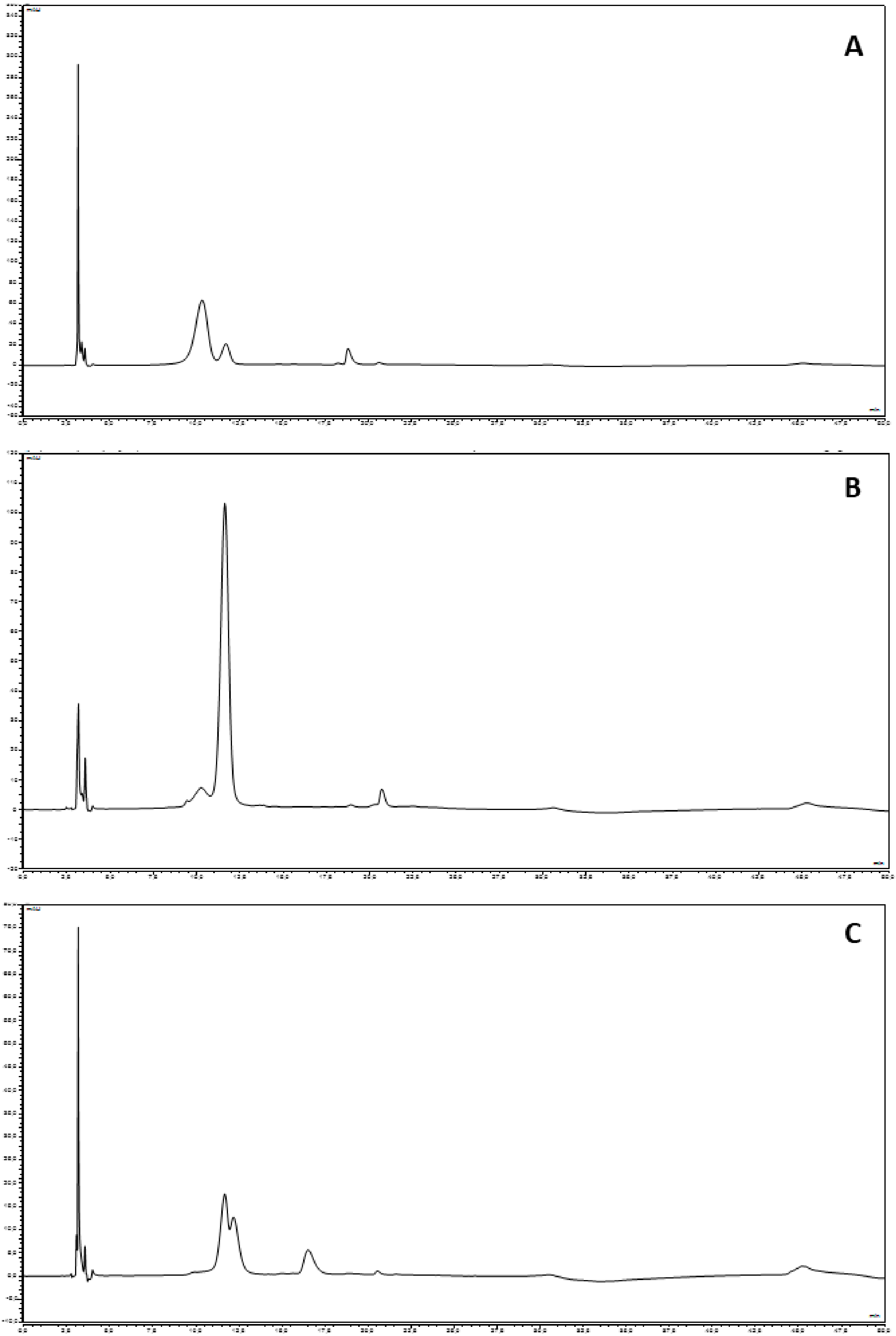

Table S3. Key to populations of *Phyteuma* included in this work.

Key to populations of *Phyteuma* in Slovenia

1. Flowers white or light-violet. Colorimetric parameters: L>45, a<2, b<45, C<35, h<280 2

    2. Anthocyanins absent or in traces .... **PSS**

    2'. Anthocyanins present (D3R, C3R, Po3G) .... **PSC**

1'. Flowers violet, blue or purple. Colorimetric parameters: L<45, a>2, b>45, C>35, h>280 3

    3. Anthocyanins D3R:C3R ratio 1:100. Presence of neochlorogenic acid and isorhamnetin malonyl-glucoside.................... **PP**

    3'. Anthocyanins D3R:C3R ratio 1:1 to 1:25. Absence of neochlorogenic acid and isorhamnetin malonyl-glucoside 4

        4. Presence of PlRd. Colorimetric parameters: a>8, h<300 .... **PO-V and PO-DV**

        4'. Absence of PlRd. Colorimetric parameters: a<6, h>300 .... **PO-P**
